# Antibacterial Soy Protein Isolate Prepared by Quaternization

**DOI:** 10.3390/ijms23169110

**Published:** 2022-08-14

**Authors:** Qi Dong, Jingwen Lei, Hanjian Wang, Meifang Ke, Xiao Liang, Xindi Yang, Hui Liang, Céline Huselstein, Zan Tong, Yun Chen

**Affiliations:** 1 Department of Biomedical Engineering and Hubei Province Key Laboratory of Allergy and Immune Related Disease, TaiKang Medical School (School of Basic Medicine Sciences), Wuhan University, Wuhan 430071, China; 2 Research Center for Medicine and Structural Biology, TaiKang Medical School (School of Basic Medicine Sciences), Wuhan University, Wuhan 430071, China; 3 UMR 7365 CNRS, Medical School, University of Lorraine, 54505 Nancy, France

**Keywords:** soy protein isolate, quaternization, antibacterial property, wound healing

## Abstract

Soy protein isolate (SPI) is green, high-yield natural plant protein, which is widely applied in industry (packing material and adhesives) and tissue engineering. It is meaningful to improve the antibacterial property of soy protein isolate to fabricate versatile safe products to meet people’s requirements. In this study, quaternized soy protein isolate (QSPI) was synthesized by the reaction between 2,3-epoxypropyltrimethylammonium chloride (EPTMAC) and SPI. The positive charged (17.8 ± 0.23 mV) quaternary ammonium groups endow the QSPI with superior antibacterial properties against multiple bacteria in vitro and in vivo. Notably, QSPI maintains its good biocompatibility and promotes bacterial-infected wound healing in rat models. Furthermore, QSPI possesses superior water solubility in a broad pH range than raw SPI. Altogether, this soy protein isolate derivative with antibacterial property and superior water solubility may extend the application of SPI in industry and tissue engineering.

## 1. Introduction

Soy protein isolate (SPI) is a natural and nutritious plant-based protein with a protein content higher than 90% [1]. Because of its ready availability, low price, and renewable and degradable properties, SPI has been extensively applied as packaging films and adhesives in the industry to replace petroleum feedstocks [2,3]. Moreover, SPI possesses biocompatibility, non-toxicity of degradation products, and contains abundant functional amino acids that are beneficial for tissue regeneration [4]. Thus, SPI is also widely used as a biomaterials for tissue engineering. As people’s requirements for the safety of the living environment increase, the new packaging films should have a good antibacterial property to extend the shelf life of food [5,6]; the new adhesives should possess antibacterial properties to improve the mildew-resistant property and durability [7]. However, the polysaccharides and proteins in SPI are susceptible to corrosion by bacteria and fungi. Thus, many antibacterial agents such as natural compounds, curcumin, tannins, carvacrol; nanoparticles, zinc oxide, silver nanoparticles; and quaternized polymers have been incorporated into SPI-based materials to fabricate antibacterial, environmentally friendly industrial materials [8,9,10,11]. For SPI-based biomaterials, a good antibacterial property is especially important. Therefore, some antibacterial agents such as antibiotics, nanosilver, and quaternized chitosan were introduced into SPI-based biomaterials to improve the antibacterial property of biomaterials, especially for the repair of some infected wounds [12]. If endowed with the antibacterial property itself, SPI could be used to fabricate even more versatile products for practical application.

To fabricate SPI-based materials, some harsh dissolving conditions (pH >10; enzyme treatment; polar solvents) have been employed to dissolve SPI due to the poor solubility of SPI, which limits its further application in cooperation with other materials for the fabrication of composite materials [13,14,15]. For example, Chen et al. prepared a variety of SPI-based composite sponge conduits by dissolving SPI in a strong alkaline solution [16]. Qi et al. used papain partial hydrolysis of SPI to improve its water solubility [17]. Therefore, a simple modification to improve the antibacterial property and water solubility of SPI will open a new avenue for the application of SPI in industry and tissue engineering.

Quaternization modification not only endows antibacterial properties to the substrate but also improves hydrophilicity by introducing positive charged quaternary ammonium groups. For example, quaternized chitin displays an excellent antibacterial ability against *Escherichia coli* and *Staphylococcus aureus* [18]. Meanwhile, the positive charged quaternized ammonium groups reconstruct the inter- and intra-molecular hydrogen bond of the chitin chains, decreasing the degree of crystallinity and increasing the solubility of the chitin. However, quaternized soy protein isolate (QSPI) with an antibacterial property has not been reported.

**Figure 1:**
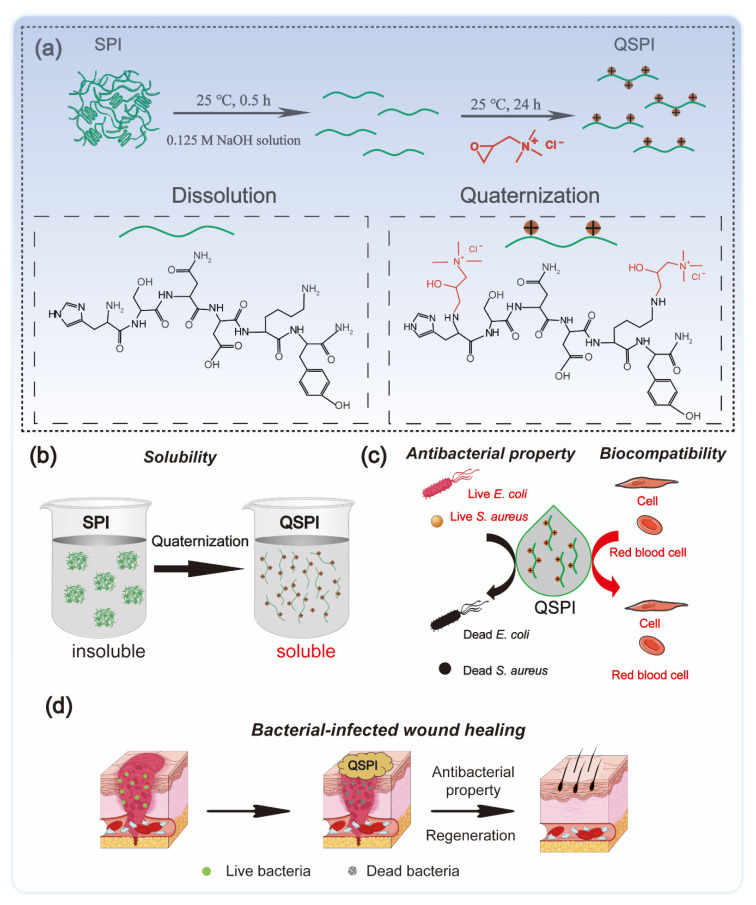
Schematic illustration of the fabrication and application of quaternized soy protein isolate. (**a**) The synthetic method of QSPI. (**b**) The water solubility of QSPI. (**c**) The antibacterial property and biocompatibility of QSPI. (**d**) The therapeutic process of bacterial-infected wound healing by QSPI.

In this work, QSPI was synthesized by the reaction between 2,3-epoxypropyltrimethylammonium chloride (EPTMAC) and SPI (Figure 1a). The antibacterial property and water solubility of QSPI were evaluated (Figure 1b,c). The potential application as an antibacterial reagent for infected wound repair was investigated (Figure 1d).

## 2. Results and Discussion

### 2.1. Fabrication and Characterization of QSPI

**Figure 2:**
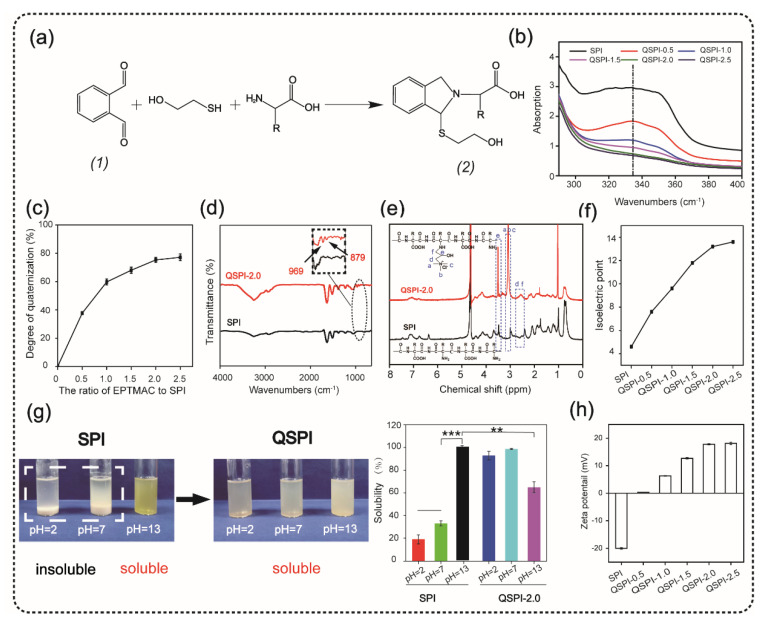
Synthesis and characterization of quaternized soy protein isolate (QSPI). (**a**) The reaction principle of OPA reagent and compound (2) has a marked absorption peak at 336 nm in the UV–vis absorption spectra (**b**). (**c**) The degree of quaternization of the QSPI-n. (**d**) The FTIR spectra of SPI and QSPI-2.0. (**e**) The ^1^HNMR spectra of SPI and QSPI-2.0. (**f**) The isoelectric point of SPI and QSPI-n. (**g**) The water solubility of SPI and QSPI-n in aqueous solution with pH = 2, 7, and 13. The water solubility of SPI in solution with pH = 13 as control. (**h**) The zeta potential of QSPI-n at pH = 7. ** *p* < 0.01 and *** *p* < 0.001.

The quaternized soy protein isolate (QSPI) was synthesized in a two-step reaction between SPI and EPTMAC, as shown in Figure 1a. Firstly, SPI was dissolved in NaOH solution (0.125 M) and stirred for 30 min at 25 °C to disrupt the inter- and intra-molecular bonds between SPI chains. Secondly, EPTMAC was added, and the mixture was stirred for 24 h at 25 °C to produce the QSPI. The *w*/*w* ratio of EPTMAC to SPI in the reaction was the primary factor influencing the degree of quaternization of QSPI. Therefore, a series of *w*/*w* ratios of EPTMAC to SPI (0.5, 1.0, 1.5, 2.0, 2.5) was set. The degree of quaternization was determined by the OPA reagent (Figure 2a). The –NH_2_ in SPI or QSPI without quaternization can react with the OPA reagent and exhibits a characteristic absorption peak at 336 nm in the UV–vis absorption spectra (Figure 2b). Thus, a higher absorption at 336 nm means that more –NH_2_ in SPI was not quaternized. A high absorption at a wavelength of 336 nm was observed in SPI, while the absorption of QSPI-n at 336 nm reduced from 2.93 in SPI to 1.83 in QSPI-0.5 and 0.67 in QSPI-2.5 due to more moles of –NH_2_ of SPI reacting with EPTMAC. The degree of quaternization was calculated and is shown in Figure 2c. As the ratios (*w*/*w*) of EPTMA to SPI increased from 0.5 to 2.5, the degree of quaternization increased from 37.69% of QSPI-0.5 to 77.15% of QSPI-2.5. When the ratio exceeds 2.0 (75.29%), the degree of quaternization changes little because the reaction tends to saturate. Therefore, the ratio of 2.0 was selected for the subsequent experiments. To further confirm the formation of the QSPI, the molecular structure of the QSPI was analyzed by Fourier transform infrared (FTIR) and ^1^HNMR. In the FTIR spectrum of SPI (Figure 2d), the characteristic absorption peaks of O–H stretching, amide I band (C=O stretching), amide II band (N–H bending), and amide III band (C–N stretching) presented at 3274 cm^−1^, 1640 cm^−1^, 1532 cm^−1^, and 1236 cm^−1^ in the spectrum of SPI and QSPI [19]. After quaternization, two new peaks appeared at 969 cm^−1^ and 879 cm^−1^ in the spectrum of QSPI. The peak at 969 cm^−1^ is for the C–N stretching vibration from –N^+^(CH_3_)_3_, and 879 cm^−1^ is for the vibration of N–H. These new characteristic absorption peaks indicated that the quaternary ammonium groups were successfully grafted onto the side chain of SPI. The structure of SPI in NaOH/D_2_O solution and QSPI-2.0 in D_2_O were further analyzed by ^1^HNMR (Figure 2e). The characteristic peaks of QSPI at chemical shift 3.14 ppm, 2.67 ppm, and 3.52 ppm were attributed to H in the –N^+^(CH_3_)_3_ group, methylene and methine, respectively [20]. These results revealed that QSPI was successfully synthesized.

The isoelectric point is a remarkable physical characteristic of protein. The isoelectric point of QSPI-n is shown in Figure 2f. The isoelectric point increased from 4.6 of SPI to 13.2 of QSPI-2.0 and 13.6 of QSPI-2.5. As the *w*/*w* ratios of EPTMAC to SPI increased, more positive charged quaternary ammonium groups were grafted onto the SPI, resulting in the increase in the isoelectric point. Proteins have the lowest solubility at the isoelectric point and have good water solubility in solutions with a pH far from the isoelectric point [7]. The isoelectric points of QSPI-2.0 and QSPI-2.5 are closer to the pH extreme than other groups, so they may have better water solubility in a broader range of pH.

Water solubility plays an important role in the application of protein biomaterials. The photograph of SPI and QSPI-2.0 in the solution with pH = 2, 7, and 13 is shown in Figure 2g. SPI exhibited aggregation and sedimentation at pH = 2 and 7, and a homogeneous transparent yellow at pH = 13. In contrast, QSPI-2.0 displayed homogeneous transparent at pH = 2, 7, and 13. The quantitative analysis of water solubility showed that SPI displayed good water solubility at pH = 13 (100%), and poor water solubility at pH = 2 (19.11 ± 3.93%) and pH = 7 (33.02 ± 2.45%) (Figure 2g). Interestingly, QSPI-2.0 was almost 100 % soluble at pH = 2, 7 and partially dissolved at pH = 13 (64.90 ± 4.84%), close to the isoelectric point (13.2) of QSPI-2.0. QSPI-2.0 had a better solubility than SPI in a broader range of pH.

The zeta potentials of the SPI and QSPI-2.0 are shown in Figure 2h. With the increase in positive charged quaternary ammonium groups, the zeta potentials increased from −20 ± 0.33 mV of SPI to 17.8 ± 0.23 mV of QSPI-2.0 and 18.1 ± 0.46 mV of QSPI-2.5 at pH = 7. Quaternarization may change the zeta potential of the target protein. For example, Zhang et al. [18] synthesized a quaternized chitin by the reaction between chitin and EPTMAC. After quaternization, the zeta potential of quaternized chitin increased from 0.28 ± 0.03 mV of chitin to 30.57 ± 0.99 mV of quaternized chitin. In summary, QSPI was successfully synthesized, and QSPI-2.0 has good water solubility in a broad range of pH.

### 2.2. Antibacterial Property Evaluation of QSPI-2.0

**Figure 3:**
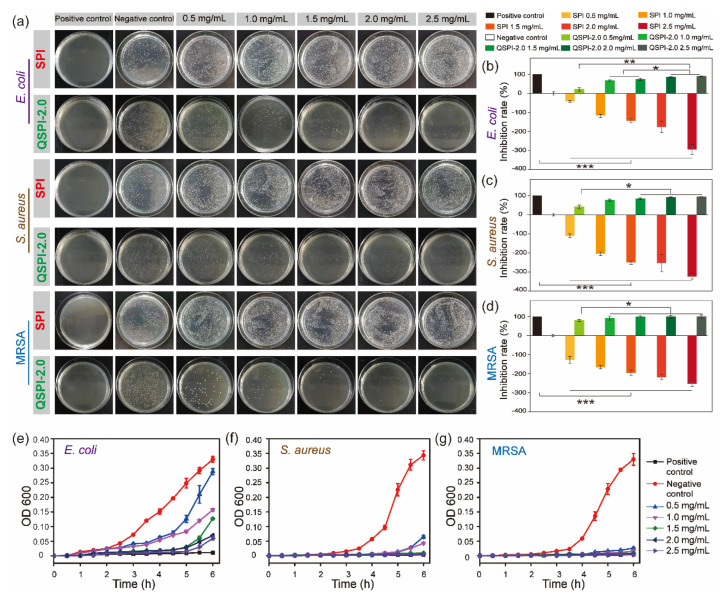
The antibacterial property of SPI and QSPI-2.0. (**a**) Agar plate images of CFU test for antibacterial properties of SPI and QSPI-2.0 against *E. coli*, *S. aureus* and MRSA. The statistical analysis results of the CFU test of *E. coli* (**b**); *S. aureus* (**c**); and MRSA (**d**). The results of proliferation assay of QSPI-2.0 with different concentrations against *E. coli* (**e**); *S. aureus* (**f**); and MRSA (**g**). * *p* < 0.05; ** *p* < 0.01 and *** *p* < 0.001.

Quaternary ammonium compounds are widely used to kill bacteria. The strong positive charges of quaternary ammonium groups may endow QSPI with antibacterial properties. Therefore, three common bacteria, *E. coli* (Gram-negative), *S. aureus* (Gram-positive), and MRSA were used to evaluate the antibacterial property of the raw SPI and QSPI-2.0. In the colony-forming units (CFU) testing, SPI exhibited no antibacterial property (Figure 3a). Meanwhile, the effects of promoting bacterial growth became more obvious as the concentration of SPI increased. The capacity of promoting the growth of bacteria limits the application of SPI. In contrast, QSPI-2.0 exhibited significant antibacterial properties. The statistical analysis results showed that the inhibition rate increased with the increase in QSPI-2.0 concentration, from 20.86 ± 3.16% (0.5 mg/mL) to 89.17 ± 1.15% (2.5 mg/mL) for *E. coli* (Figure 3b), from 40.86 ± 9.31% (0.5 mg/mL) to 94.17 ± 1.15% (2.5 mg/mL) for *S. aureus* (Figure 3c), and from 80.24 ± 5.80% (0.5 mg/mL) to 99.79 ± 9.26% (2.5 mg/mL) for MRSA (Figure 3d). The same tendency was also observed in the results of the proliferation assay. As shown in Figure 3e–g, the proliferation rates of the three bacteria remarkably declined with the increase in QSPI-2.0 concentration. The antibacterial property of QSPI-2.0 is probably due to the positive charges of quaternary ammonium groups. The positive charged groups interact with the negative charged cytomembrane of bacteria, damaging the cytomembrane and killing bacteria [21,22,23]. Thus, these results demonstrated that the QSPI-2.0 had a good antibacterial property. QSPI can be used as an antimicrobial agent for the fabrication of antibacterial, environmentally friendly SPI-based packaging films and adhesives.

### 2.3. Biocompatibility Evaluation of QSPI-2.0

**Figure 4:**
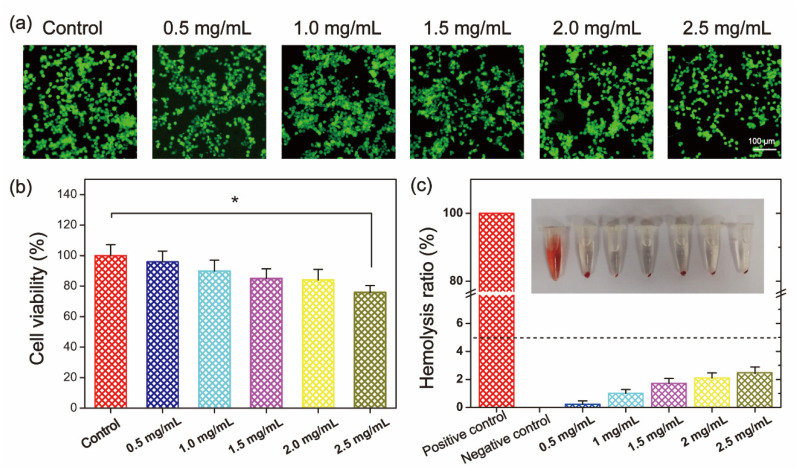
Biocompatibility evaluation of QSPI-2.0. (**a**) Live/dead cell staining of L929 cells after treatment with 0.5–2.5 mg/mL QSPI-2.0 for 48 h. (**b**) Cell viability of L929 cells after treatment with 0.5–2.5 mg/mL QSPI-2.0 for 48 h. (**c**) Hemolysis assay of QSPI-2.0. * *p* < 0.05.

The biocompatibility of QSPI was evaluated by cytocompatibility and hemocompatibility in vitro. Firstly, the cytocompatibility of the QSPI-2.0 was accessed by live/dead staining and the (3- (4,5-dimethyl-2-thiazoLyl) -2,5-diphenyl tetrazolium bromide (MTT) assay. L929 cells treated with different concentrations of QSPI-2.0 were stained with calcein-AM (green) for live cells and propidium iodide (PI) for dead cells (Figure 4a). Almost all cells exhibited normal shapes and stained green demonstrating no significant cytotoxicity of the QSPI-2.0. In the MTT assay, the cell viability exhibited no significant difference when the concentrations of QSPI-2.0 increased from 0.5 to 2.0 mg/mL (Figure 4b). However, the cell viability slightly decreased after treatment with a high concentration (2.5 mg/mL) of QSPI-2.0. These data revealed that QSPI had excellent cell biocompatibility.

Furthermore, the hemocompatibility of QSPI-2.0 was tested by a hemolysis experiment (Figure 4c). After being incubated with QSPI-2.0, blood displayed light yellow after centrifugation, which is similar to the blood treated with the negative control (PBS). In contrast, Triton-treated blood as the positive control was bright red. Quantitative evaluation results indicated that when the concentrations of QSPI-2.0 increased from 0.5 to 2.5 mg/mL, the hemolysis ratios were slightly increased from 0.233 ± 0.096% to 2.40 ± 0.49%, which were less than the permissible limit (5%). These results revealed that QSPI-2.0 had good cytocompatibility and hemocompatibility, which may have potential as biomaterials [24].

### 2.4. In Vivo Antibacterial Property and Wound Healing Evaluation of QSPI-2.0

**Figure 5:**
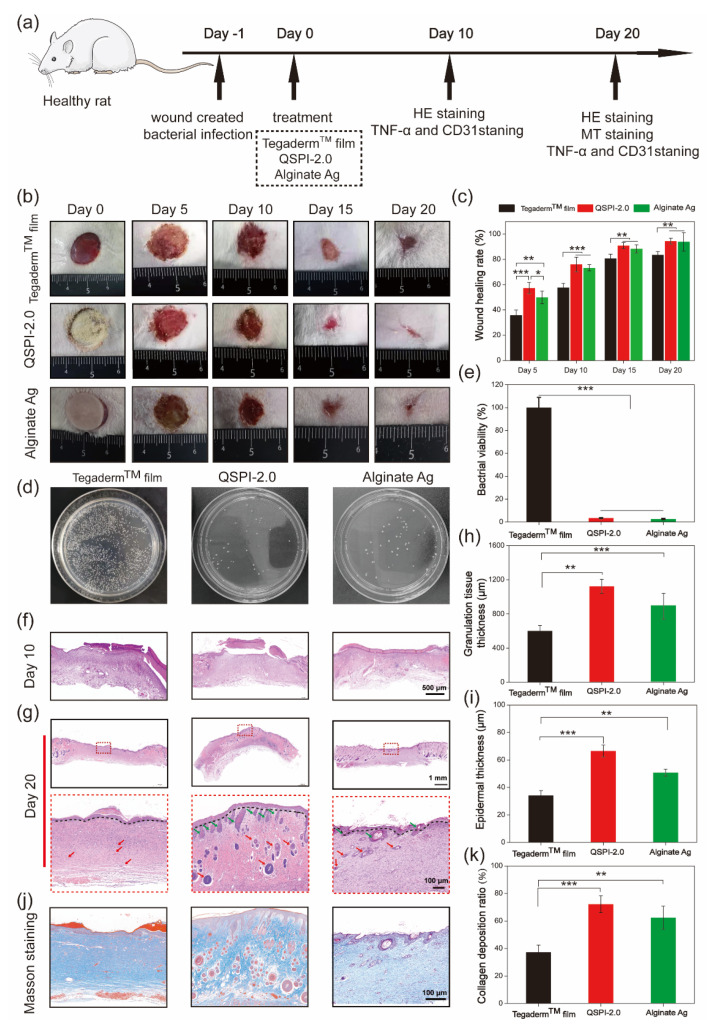
Effects of the QSPI-2.0 in a rat infected wound healing model. (**a**) The schematic diagram illustrates the establishment and treatment of the bacteria-infected wound. (**b**) Representative images of the bacteria-infected wound healing. (**c**) Quantification of wound healing rate. (**d**) Photographs of CFU test of bacteria derived from wound sites on Day 3 after different treatments. (**e**) Bacterial viability was calculated according to the CFU test. Images of histological analysis for wound regeneration on Day 10 (**f**) and Day 20 (**g**). Black lines point to the boundary of epidermis and dermis; green arrows point to the hair follicles and red arrows point to blood vessels. Statistical results of (**h**) granulation tissue thickness of the regenerating skin on Day 10 and (**i**) epidermal thickness of the regenerated skin tissue on Day 20. (**j**) Masson trichrome staining images of the regenerated skin tissue. (**k**) Quantification of the collagen deposition ratio in each group on Day 20. * *p* < 0.05; ** *p* < 0.01 and *** *p* < 0.001.

To further examine the antibacterial ability and promotion of infected wound healing of QSPI-2.0 in vivo, an MRSA-infected skin wound model was established in rats. QSPI, Alginate Ag, and Tegaderm^TM^ film were used as wound dressings (Figure 5a). Representative photos of the wound sites in different groups at scheduled time intervals are shown in Figure 5b. The QSPI-2.0 and Alginate Ag showed a significantly higher wound healing rate than the Tegaderm^TM^ film. More specifically, the QSPI-2.0 group and the Alginate Ag group led to 76.03 ± 5.76% and 73.35 ± 2.45% wound healing rates on Day 10, which were significantly higher than the Tegaderm^TM^ film (57.65 ± 3.45%) (Figure 5c). On Day 20, the wound treated with QSPI-2.0 was almost healed with the wound healing rate closed to 100%, while the wound healing rate in the Alginate Ag group and Tegaderm^TM^ film were approximately 90.38 ± 7.25% and 83.69 ± 2.68%. These results indicate that the QSPI-2.0 could promote infected wound healing.

To further evaluate the antibacterial property of QSPI-2.0 in vivo, the amounts of surviving bacteria at the wound site of different groups were evaluated on Day 3 after treatment. As shown in Figure 5d, a few colony units were observed in QSPI-2.0 and Alginate Ag groups, while the Tegaderm^TM^ film group exhibited poor bacterial property with lots of colony units. The QSPI-2.0 group and the Alginate Ag group could efficiently eliminate bacteria in vivo and displayed only 3.42 ± 0.44% and 2.64 ± 0.55% bacterial viabilities compared with Tegaderm^TM^ film (Figure 5e). These results indicated that the bacteria infection in the wound tissue could be effectively inhibited by QSPI-2.0 due to its excellent antibacterial property.

The wound healing effects were further analyzed by histological analyses. The H&E and Masson’s trichrome staining of the wound tissue on Day 10 and Day 20 were performed. As shown in Figure 5f, less inflammatory cells in the QSPI-2.0 group and Alginate Ag group were observed compared with the Tegaderm^TM^ film group on Day 10, which was probably due to QSPI-2.0 and Alginate Ag inhibiting bacterial infection-induced inflammation [25]. Granulation tissue formation is a critical stage during the wound healing process, where the injury is filled with a matrix of fibrous connective tissue and blood vessels. Thicker granulation tissue may create a better framework for the other cells to grow, fill in the wound and restore skin function. Thus, the granulation tissue was observed by H&E staining, and its thickness was calculated on Day 10 (Figure 5h). The thickness of the granulation tissue of the QSPI-2.0 group was significantly higher than those of the other groups, suggesting a better wound regeneration environment of the QSPI-2.0 group. The epidermal thickness is a key index to evaluate the wound healing effects at the end stage of wound repairing [26]. Thus, the epidermal thickness was observed by H&E staining on Day 20 after treatment (Figure 5g). The thickest epithelium (66.49 ± 4.26 μm) of the QSPI-2.0 group among the three groups, indicating the best re-epithelialization effect (Figure 5i). In addition, the regenerated tissue of the QSPI-2.0 group exhibited better organized structures including more hair follicles and blood vessels than those in the Alginate Ag group and Tegaderm^TM^ film group.

Moreover, collagen deposition emerges during the ECM remodeling phase of the wound healing process, which is crucial for improving ECM formation [27]. Masson trichrome staining was used to evaluate the collagen levels in the newly formed connective tissue at wound sites. The images of Masson trichrome staining (blue area representing collagen fiber) on Day 20 were shown in Figure 5j. More dense and oriented collagen fibers were observed in the QSPI-2.0 group compared with the Tegaderm^TM^ film group. Quantitative analysis demonstrated that the QSPI-2.0 group had the highest collagen index (relative intensity of collagen deposition) among the three groups, indicating that the deposition of ECM was remarkably increased in QSPI-2.0 treated wounds (Figure 5k). All the data demonstrated that QSPI-2.0 had a superior capacity of promoting infected wound healing.

**Figure 6:**
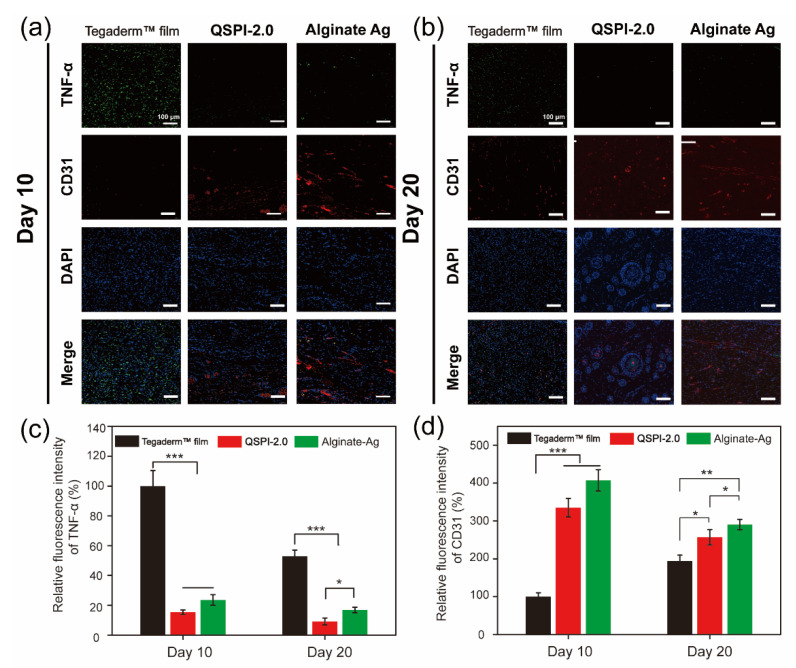
Immunofluorescence images of the regenerated wound tissue. Immunofluorescence staining against TNF-α (green) and CD31 (red) on Day 10 (**a**) and Day 20 (**b**); Statistical analysis of TNF-α (**c**) and CD31 (**d**) relative fluorescence intensity. * *p* < 0.05; ** *p* < 0.01 and *** *p* < 0.001.

The continuous bacterial infection-induced prolonged inflammatory phase is the greatest obstacle to wound healing in the early healing stage [28]. Therefore, the expression of the typical inflammatory factor tumor necrosis factor (TNF-α) in the wound site was assayed by immunohistochemistry on Day 10 and 20 (Figure 6a,b) and quantified in Figure 6c. Although the ratios of TNF-α positive cells decreased over time in all three groups, the Tegaderm^TM^ film group showed a severe inflammatory response with the highest expression levels of TNF-α. In contrast, the QSPI-2.0 and Alginate Ag groups exhibited lower levels of TNF-α expressions on Day 10 and Day 20, suggesting less inflammation or infection in the wound sites. These data are consistent with the results that QSPI-2.0 had antibacterial properties, inhibited bacterial infection in the wound sites, and less inflammation was observed in the H&E staining of regenerating skin tissues.

On the other hand, neovascularization supports cell migration and proliferation in the newly forming tissue, which is essential for re-epithelialization and chronic wound healing [29,30,31]. Therefore, the formation of blood vessels in the wound site was detected using the immunofluorescent staining of endothelial cell specific protein marker, CD31. Red fluorescence was hardly detectable in the Tegaderm^TM^ film group. However, QSPI-2.0 and Alginate Ag groups exhibited high levels of CD31 expression. The neovascularization in the QSPI-2.0 group appeared to have grossly more CD31 expression than the Tegaderm™ film on day 10 (Figure 6d). Besides, the CD31 expression level was lowest in the Tegaderm™ film group and there was a significant difference between the QSPI-2.0 and Alginate Ag groups on Day 20. Additionally, more mature round capillaries were found in the QSPI-2.0 group, indicating the promoting angiogenesis effects of QSPI-2.0. In summary, QSPI-2.0 effectively accelerates wound healing by simultaneously reducing bacterial infection-induced inflammation and promoting angiogenesis.

## 3. Materials and Methods

### 3.1. Materials

Soy protein isolate (Mw: 205 KDa, protein content: 95%) was purchased from DuPont Protein Technology (Luohe, China), and vacuum-dried for 24 h before use. 2,3-epoxypropyltrimethylammonium chloride (EPTMAC), sodium hydroxide, acetic acid, and ampicillin (Amp) were supplied by Aladdin (Shanghai, China). Ethanol and o-phthalaldehyde were obtained from Guoyao Chemical Company (Shanghai, China).

### 3.2. Synthesis of Quaternized Soy Protein Isolate

The synthesis method of quaternized soy protein isolate (QSPI) is as follows: 6 g SPI was suspended in 100 mL 0.125 M NaOH aqueous solution, followed by stirring for approximately 30 min at 25 °C to obtain a homogeneous solution. A specified amount of EPTMAC (3, 6, 9, 12, 15 g) dissolved in 10 mL deionized water was added slowly into the SPI solution, and the mixture reacted at 25 °C for 24 h. Next, the supernatant was collected by centrifugation at 5000 rpm for 20 min, neutralized to neutral with acetic acid, precipitated in 500 mL ethanol, and then filtered. The filter cake was dried at 50 °C for 24 h to obtain quaternized soy protein isolate (QSPI). According to the amount of EPTMAC participated in the reaction, a series of quaternized soy protein isolates were synthesized and coded as QSPI-n (n = 0.5, 1.0, 1.5, 2.0, 2.5), where “n” represents the ratio (*w*/*w*) of EPTMAC to SPI.

### 3.3. Chemical and Physical Characterization of QSPI

The degree of quaternization was determined using the OPA (o-phthalaldehyde) method [32]. The OPA reagent was freshly prepared by adding OPA (40 mg), methanol (1 mL), β-mercaptoethanol (100 μL), and 20% wt of SDS (2.5 mL) into 0.1 M sodium borate buffer (25 mL, pH 9.75). The mixture was diluted to 50 mL. Subsequently, 4 mL OPA reagent was reacted with 200 μL 0.4% QSPI solution at 37 °C for 2 min. The absorption at 336 nm was measured by a UV spectrophotometer (Shimadzu, Kyoto, Japan).

The degree of quaternization was calculated according to the equation:

Degree of quaternization (%) = [(A_0_ − A_s_)/A_0_] × 100% (1)

where: A_0_ refers to the absorbance of the SPI, As refers to the absorbance of the QSPI.

The ^1^HNMR spectra of QSPI and SPI were recorded using a Bruker Avance 500 MHz. The Fourier transform infrared (FTIR) spectra were analyzed from 4000 to 500 cm^−1^ (PerkinElmer System 2000 spectrometer, PerkinElmer, Waltham, MA, USA).

The isoelectric point was measured by Nano-ZS ZEN3600 (Malvern Instruments, Malvern, UK). After the QSPI dissolved in deionized water at a 1 mg/mL concentration, the pH of the solutions was adjusted from 1, 2, 3, to 14, and the zeta potential was measured by Nano-ZS ZEN3600. When the zeta potential is 0, the corresponding pH is the isoelectric point.

After the QSPI was dissolved in deionized water at pH = 2, 7, and 13 with a concentration of 10 mg/mL, the water solubility of the QSPI was measured by the UV spectrophotometer at the absorbance of 280 nm [33]. The SPI could be dissolved thoroughly when the pH ≥ 11 and the absorption of QSPI in deionized water with pH = 13 was used as control [34].

The water solubility was calculated according to the equation:

Water solubility (%) = (I_s_/I_13_) × 100% (2)

where: I_13_ refers to the absorbance of the SPI in the solution of pH = 13, Is refers to the absorbance of the QSPI or SPI.

### 3.4. Antibacterial Property Evaluation of QSPI

The antibacterial property of QSPI against Gram-negative bacteria Escherichia coli (*E. coli*), Gram-positive bacteria Staphylococcus aureus (*S. aureus*) and Methicillin-resistant Staphylococcus aureus (MRSA) was assessed by the proliferation assay and colony forming units (CFU) testing [35,36]. For the proliferation assay, 10 mL of sterilized QSPI solution (PBS as solvent) with increasing concentrations (0, 0.5, 1.0, 1.5, 2.0, 2.5 mg/mL) was added to centrifugation tubes, followed by the addition of 100 μL of bacterial suspension (10^6^ CFU/mL). Bacterial suspension with antibiotic (ampicillin) was used as the positive control, and bacterial suspension with 0 mg/mL of QSPI was used as the negative control. Then the centrifugation tubes were incubated at 37 °C for 12 h. 100 μL mixture was added into a 5 mL LB medium and incubated at 37 °C. Then, 200 μL LB medium was taken from each group at each time interval, and its OD value was measured at the wavelength of 600 nm via a microplate reader (Spectra Max M2). The suspension turbidity at 600 nm is proportional to the number of bacteria. For the CFU test, after the bacteria co-cultured with QSPI, the mixture was step wisely diluted with PBS, and then cultured on agar plates. After incubation at 37 °C for 12 h, the bacterial colonies were photographed and counted. Each experiment was repeated three times. The inhibition rate was calculated with the following equation:

Inhibition rate (%) = (C_N_ − C_S_)/(C_N_ − C_p_) × 100% (3)

where C_N_, C_p_, and C_S_ stand for the average colony counts of the negative control, positive control, and QSPI groups, respectively.

### 3.5. Cytocompatibility and Hemocompatibility of the QSPI

Fibroblast cell line (L929) was used to evaluate the cytocompatibility of QSPI by 3- (4,5-dimethyl-2-thiazolyl) -2,5-diphenyl-2-H-tetrazolium bromide (MTT) assay and live/dead assay. L929 cells were seeded into a 96-well plate with 2 × 10^3^ cells/well for 24 h at 37 °C. Then, the cell culture medium was updated with 200 μL serial dilutions of QSPI (0.5, 1.0, 1.5, 2.0, 2.5 mg/mL) in the culture medium for another 48 h and without QSPI as control. Then, the culture medium was replaced by 20 μL MTT solution and incubated for 4 h. Finally, the MTT solution was abandoned and 200 μL of DMSO was added to each well to dissolve the formazan crystals. The absorbance at 490 nm was measured via a microplate reader (SpectraMax M2, Molecular Devices, San Jose, CA, USA). The cell viability of L929 cells was analyzed using the following equation:

Cell viability (%) = (A_S_ − A_B_)/(A_N_ − A_B_) × 100% (4)

where A_S_, A_N_, and A_B_ represent the absorbance of the sample, negative control, and blank control at a wavelength of 490 nm, respectively.

Live/dead staining was conducted with L929 cells seeded in 24-well plate with 2 × 10^4^ per well. After cocultured with 1 mL serial dilutions of QSPI solution (0.5, 1.0, 1.5, 2.0, 2.5 mg/mL) at 37 °C for 48 h, the L929 cells were stained with 500 μL of calcein-AM/propidium iodide dye at room temperature for 30 min. The cells were observed via a fluorescent microscope for the green (492 nm) and red (545 nm) fluorescence.

The hemolysis of QSPI was analyzed by incubating QSPI with rabbit’s red blood cells. The red blood cells were obtained via centrifugation of whole blood at 3500 rpm for 10 min and diluted with PBS to 5% (*v*/*v*). The 0.5 mL red blood cells suspension was added into 0.5 mL of gradient concentrations of QSPI solution (0.5, 1.0, 1.5, 2.0 mg/mL) and incubated at 37 °C for 1 h. The red blood cells suspension incubated with PBS was used as the negative control, and 0.1% Triton X-100 solution was used as the positive control. The mixture was further centrifuged at 3500 rpm for 5 min, and 200 μL supernatant was transferred to a 96-well plate. The absorbance was measured at 540 nm via a UV spectrometer (SpectraMax M2). The hemolysis rate of QSPI is estimated by the following equation:

Hemolysis ratio (%) = (A_S_ − A_N_)/(A_P_ − A_N_) × 100% (5)

where A_S_, A_N_, and A_P_ represent the absorbance of QSPI and the negative and positive controls, respectively.

### 3.6. In Vivo Bacterial-Infected Wound Healing Assay

All the rats used in the experiments were supplied by ABSL-III Center for Animal Experiment at Wuhan University and handled following Wuhan University Animal Care Committee. The bacterial-infected wound healing assay of QSPI-2.0 was analyzed via a rat MRSA-infected full-thickness skin defect model. Thirty SD rats (280–300 g, 7–8 weeks) were anesthetized by an intraperitoneal injection of 1% sodium pentobarbital and shaved on the dorsum. Then, two round full thickness wounds (diameter: 15 mm) were inflicted on the dorsum of each rat, and the 100 µL suspension containing MRSA (1 × 10^7^ CFU mL^−1^) was immediately dropped onto the wounds for 24 h to establish the MRSA-infected full thickness wound model. All these rats were randomly divided into three groups with ten rats in each group and treated with Tegaderm^TM^ film (a commercial film dressing) as the negative control, commercial antibacterial dressing Biatain Alginate Ag sponge (coded as “Alginate Ag” in this study) as the positive control, and QSPI-2.0, respectively. The wound sites were photographed on Day 0, 5, 10, 15, and 20. The wound area was analyzed via Image-J. The wound healing rate was calculated by the following formula:

Wound healing rate (%) = (S_0_ − S_t_)/S_0_ × 100% (6)

where S_0_ is the initial wound area, and S_t_ is the wound area at a specific time point.

To evaluate the antibacterial property in vivo, the infected wound tissues were collected after 3 days of treatment and homogenized with 5 mL PBS to separate the bacteria. Then, 200 μL of PBS containing bacteria was plated on the LB agar for 12 h. The viable bacteria were photographed and measured by counting the colony-forming units.

For histological analysis, the wound tissues were harvested on Day 10 and 20 after treatments, and fixed in 4% formaldehyde, embedded in paraffin, and sectioned into 3–4 μm thickness sections. Haematoxylin-Eosin (H&E) and Masson’s staining were performed for histological analysis. For quantitative analysis of the histopathological changes, the thickness of granulation tissue at day 10, the thickness of the epidermis, and the ratio of the collagen-occupied area at day 20 were calculated based on the staining images by Image-J.

Inflammation on Day 10 and angiogenesis on Day 20 after wound treatment were, respectively, determined by TNF-α and CD31 immunofluorescence staining. The results were photographed by inverted fluorescence microscopy (DMi8, Leica, Germany), and the fluorescence intensity was determined with Image-J.

### 3.7. Statistical Analyses

All data were displayed as mean ± standard deviation. Statistical significance was determined by one-way ANOVA and Student’s *t*-test. *p* < 0.05 was statistically significant (* *p* < 0.05; ** *p* < 0.01; *** *p* < 0.001).

## 4. Conclusions

In this work, quaternized soy protein isolate (QSPI) was successfully synthesized via quaternization of soy protein isolate (SPI). The QSPI not only has more improved water solubility than SPI, but also shows greatly enhanced antibacterial properties and potential ability for bacteria-infected wound healing. This soy protein isolate-based derivative may open a new avenue for the applications of soy protein in industry and tissue engineering. In the future, it is very necessary to balance the water solubility, antibacterial property, and biocompatibility of QSPI by adjusting the quaternization degree or by introducing other functional groups in the SPI chains.

## Data Availability

Not applicable.
